# Bacterial Cellulose Membrane Experimentally Implanted in the Peritoneum of Wistar Rats—Inflammatory Immunoreactivity and Oxidative Stress

**DOI:** 10.3390/cimb46110697

**Published:** 2024-10-22

**Authors:** Karina Oliveira Santos, Rebecca Bertolo, Natasha Lien de Almeida Ibanez, Mônica Rodrigues Alves, Tatiana Pessoa Onuma, Gabriella Costa Ribeiro, Anna Julia de Souza Porto, Cláudio Gustavo Barbeito, Luciana Pinato, Angela Faustino Jozala, Denise Grotto, Alessandre Hataka

**Affiliations:** 1 Department of Veterinary Clinical Sciences, School of Veterinary Medicine and Animal Science, São Paulo State University (UNESP), Botucatu 18610-307, SP, Brazil; karina.o.santos@unesp.br (K.O.S.); costa.ribeiro@unesp.br (G.C.R.);; 2 Department of Pharmacy, University of Sorocaba, Sorocaba 18023-000, SP, Brazil; natasha.lien@hotmail.com (N.L.d.A.I.); angela.jozala@prof.uniso.br (A.F.J.); denise.grotto@prof.uniso.br (D.G.); 3 Laboratory of Descriptive, Experimental and Comparative Histology and Embryology, School of Veterinary Sciences, National University of La Plata, National Scientific and Technical Research Council, La Plata 1900, CP, Argentina; barbeito@fcv.unlp.edu.ar; 4 Department of Speech, Language and Hearing Sciences, São Paulo State University (UNESP), Marilia 17525-900, SP, Brazil; luciana.pinato@unesp.br

**Keywords:** bacterial cellulose, peritoneal, immunohistochemistry, oxidative stress, rat

## Abstract

Bacterial cellulose (BC) has been used for various applications; however, studies investigating the immunohistochemical characteristics of the inflammatory and scarring component in BC implanted in the peritoneum in vivo have not yet been fully described. This study aimed to evaluate the systemic and organic safety of BC through oxidative stress, blood, and serum biochemical markers, as well as the late inflammatory response in rats, using histopathology and immunohistochemistry. Forty-three rats (26 males; 17 females) received BC in the peritoneal cavity (implanted group—IG), while twenty-seven rats (12 males; 15 females) served as the control (sham group—SG). Sixty days after surgery, oxidative stress in tissues, blood biochemical markers, and histopathological and immunohistochemical analyses for lymphocytes, macrophages, collagen, and vascular response around the BC were assessed. Only one oxidative stress marker, glutathione peroxidase, was elevated in the liver of IG rats. Creatine kinase MB and lactate dehydrogenase levels were significantly lower in IG animals. Histopathological analysis showed granulomatous inflammation in 93% of IG rats, with 74% of mild intensity. Immunohistochemistry revealed a significant macrophage presence (F4/80), with CD3, CD20, and F4/80 markers indicating differences favoring macrophages. In conclusion, BC implantation in the peritoneum induces a foreign body granulomatous response with prominent macrophage presence (F4/80). Type I and III collagen were observed around the membrane, and vascularization was intense 60 days post-implantation. From a biochemical and oxidative stress perspective, BC seems to be a safe material to be used in the peritoneal cavity.

## 1. Introduction

Bacterial cellulose (BC), also known as microbial cellulose or biocellulose, is produced by aerobic bacteria, *Gluconacetobacter* species, as a key component of their biofilms [1]. Particularly, cultures of *Gluconacetobacter xylinus* produce biofilms composed of pure, non-toxic cellulose [1,2]. These bacteria secrete cellulose in the form of nano/microfibrils, which fuse into larger cellulose ribbons. In static cultures, these ribbons and associated cells form a floating nanofibrillar film that allows non-motile bacteria to thrive in the oxygen-rich surface layer of the growth medium [2].

Unlike plant cellulose, BC is pure, free from lignin, hemicellulose, pectin, and other plant compounds, and contains no animal-derived components. It also exhibits superior mechanical properties compared to plant cellulose [3,4]. Studies suggest that BC is non-toxic, does not provoke inflammation, and does not induce oxidative stress at the cellular level [5,6].

Bacterial cellulose (BC) has several key features, including biocompatibility, bio functionality, lack of toxicity, and ease of sterilization [1]. Studies have shown that BC retains humidity, preventing dehydration, traps carbon dioxide produced during the tricarboxylic acid cycle, and aids in bacterial buoyancy. This allows bacteria to survive in an aerobic environment [7].

Biocompatibility is defined as “the ability of a material to elicit an appropriate host response in a specific application” [8]. The biocompatibility and hemocompatibility of BC have been demonstrated in both in vitro/vivo studies. Generally, BC is considered highly biocompatible, provoking at most a moderate foreign body response, as observed in a dog study [8,9]. The BC membranes can also promote autolytic debridement, reduce pain, and accelerate granulation, facilitating effective wound healing [10,11]. Additionally, BC has high purity, flexibility, crystallinity density, tensile strength, water retention capacity, and a greater surface area compared to plant cellulose [2,11]. These properties make BC valuable in various industries, including in artificial fur production, cosmetics, and the food industry [12,13].

Several types of BC have been developed for diverse applications, such as vascular grafts, drug delivery systems, tissue regeneration, and scaffolds for tissue engineering in both in vitro/vivo settings [14,15,16]. Cellulose-based dressings have been explored in a variety of uses, such as artificial blood vessels, artificial bone, artificial cartilage, cancer treatment, artificial cornea, artificial meninges, wound treatment (including traumatic lesions, dehorning in bovines, diabetic wounds and pressure ulcers, venous statis, and ischemic wounds) [17,18,19,20,21,22,23,24,25,26,27].

Researchers are developing systems that replicate the complex, hierarchical structures of native tissues using both synthetic polymers and natural biopolymers, often at the nanoscale. The cellulosic nanofibril network in BC provides a biomaterial with high mechanical strength and excellent water retention [2].

Although BC has been extensively studied in vitro and in vivo, there is limited research on the inflammatory process it induces, including the roles of lymphocytes and macrophages in bio integration or implant rejection.

This study aimed to evaluate the systemic safety and potential toxicity of BC membranes implanted intraperitoneally in Wistar rats. We assessed biochemical and oxidative stress markers in blood and organs. The inflammatory response to the implanted BC membrane was analysed using histopathology and immunohistochemistry, focusing on the identification and quantification of lymphocytes (CD3 and CD20) and macrophages at the implant site. We also examined collagen types and measured vascularization (smooth muscle alpha-actin) at the implant site to determine the viability of BC as bioimplant.

The inflammatory response to the implanted BC membrane was evaluated using histopathology and immunohistochemistry techniques, including the identification and quantification of lymphocytes (CD3 or CD20) and macrophages at the implant site. Additionally, collagen fibers were typed, and vascularization (smooth muscle alpha-actin) at the implant site was measured, thus verifying the viability of BC as a bioimplant.

## 2. Materials and Methods

### 2.1. Bacterial Cellulose Membrane

Bacterial cellulose membranes were produced by cultivating *Gluconacetobacter xylinus* ATCC 53582 in Hestrin & Schramm medium using 24-well cell culture plates. Each well was inoculated with 1 mL of a culture containing approximately 10^6^ CFU. Membranes with an approximate thickness of 2 mm were grown for 4 days at 30 °C in static culture. After cultivation, membranes were washed overnight in 2% SDS, rinsed with distilled water until SDS removal, and then immersed in 1 M NaOH, under agitation at 60 °C for 1.5 h. The membranes were subsequently autoclaved in phosphate-buffered saline (PBS), at 121 °C for 15 min and stored at 4 °C [28].

### 2.2. Animals and Procedure

This study was approved by the Ethics Committee on Animal Use (CEUA) of the School of Veterinary Medicine and Animal Science, São Paulo State University (UNESP) (protocol code 226/2021) and adhered to the National Council for the Control of Animal Experimentation (CONCEA—Brazil) guidelines. Seventy Wistar rats (approx., 150 g, both sexes) were randomly divided into an implanted group (IG, 43 rats) and a sham group (SG, 27 rats) (Supplementary Material). The animals were housed individually with ad libitum access to water and food and were maintained at 23 ± 2 °C with a 12 h light/dark cycle.

Anesthesia was induced with a combination of xylazine hydrochloride (Anasedan—Ceva^®^; 7 mg/kg), ketamine hydrochloride (Dopalen—Ceva^®^; 80 mg/kg), and tramadol hydrochloride (Hipolabor^®^; 3 mg/kg) via intramuscular injection.

The abdominal region was trichotomized and disinfected. A ventral median celiotomy, with a retro-umbilical incision, was performed in order to access the abdomen. After skin and subcutaneous incisions, a 1 cm incision was performed along the linea alba. The abdominal wall was then pulled to expose the parietal peritoneum, using rat tooth forceps. A membrane with 1 × 0.5 cm was fixed to the middle third of the parietal peritoneum of the right abdominal wall with a simple isolated suture, using a black monofilament 5-0 nylon thread and a 2.5 cm CTI 3/8 tri-faceted needle (Supermedy^®^). The suture involved the parietal peritoneum and the rectus abdominis muscle, and did not involve the fascia of the muscle. The distance from the incision line to the membrane implantation site was 1 cm, so that the healing of the abdominal wall would not compromise the implanted region. The abdominal muscles and skin were closed using simple interrupted sutures, with a black monofilament 5-0 nylon thread and a 2.5 cm CTI 3/8 tri-faceted needle (Supermedy^®^), and the BC was surgically implanted at the right abdominal wall. The abdominal wall and the skin were sutured with simple separated stitches. The animals in the sham group received the same procedure detailed above, but without implanting the BC. The harvest site for this group was the site where the nylon thread was sutured to the peritoneum in the abdominal wall.

### 2.3. Biochemicals Tests and Oxidative Stress

Whole blood samples were collected in heparinized syringes and stored in tubes containing EDTA (for oxidative stress analysis), and in tubes without anticoagulants for biochemical examination (serum). Serum biochemical analyses were conducted using commercial kits from Roche Diagnóstica^®^ and Bioclin^®^ to measure albumin, ALT, AST, urea, creatinine, creatube0jubase MB (CK-MB), lactate, and lactate dehydrogenase (LDH), following the manufacturer’s protocols.

Oxidative stress was evaluated in blood, plasma, and tissues (liver and kidney). The tissues were segmented, and 250 mg of tissue was mixed with 1.5 mL of 1.15% KCl solution. The tissue was then sonicated using a Sonics Vibra-Cell^®^ Ultrasonic Atomizer for two cycles of 30 s at 35% intensity, forming the homogenate sample, being stored at −80 °C until analysis.

Reduced glutathione (GSH) determination was performed by quantifying total thiols based on the Ellman method (1959) [29]. For this, 150 µL of whole blood was hemolyzed with 100 µL of Triton X-100 and precipitated with 100 µL of 30% trichloroacetic acid. The samples were centrifuged, and the supernatants were diluted in 1 M potassium phosphate buffer pH 7.4 and reacted with 10 mM 5,5′-dithiobis-2-nitrobenzoic acid (DTNB), forming a yellow complex, which was evaluated in a spectrophotometer at 412 nm. A calibration curve with GSH was generated to calculate the GSH concentration. The results were expressed in mM/mL blood. For the homogenate, the method followed the same steps, except for hemolysis. The results are expressed in mM/g tissue.

The activity of the antioxidant enzyme GPx was determined in whole blood and homogenate, based on NADPH oxidation. The blood (or homogenate) was diluted in a solution containing reduced glutathione, glutathione reductase, NADPH, sodium azide, and 70 μL of H_2_O_2_ [30]. GPx activity was monitored for two minutes at 340 nm. By measuring the decay of NADPH absorbance, GPx activity was determined, proportional to NADPH consumption. The data were expressed in µmol NADPH/min or µmol NADPH/min/g tissue.

The method for evaluating catalase (CAT) activity was based on the decomposition of H_2_O_2_ by the enzyme over three minutes, monitored at 240 nm [31]. A 20 μL aliquot of whole blood or homogenate was diluted in 50 mM potassium phosphate buffer pH 7.0, and 70 μL of H_2_O_2_ were added to start the reaction. A variation constant (κ) was used to express the activity values in blood (κ/min or κ/min/g tissue).

Lipid peroxidation in plasma or homogenate was evaluated using thiobarbituric acid reactive substances (TBARS), adapted from the methodology of Ohkawa, Ohishi, Yagi (1979) [32]. For this, 150 µL of the sample was mixed with 50 µL of 3 M NaOH and incubated at 60 °C for thirty minutes. Subsequently, 500 µL of 6% H_3_PO_4_, 500 µL of 0.8% thiobarbituric acid (TBA), and 100 µL of 10% sodium dodecyl sulfate (SDS) were added, and the samples were incubated at 80 °C for one hour. Peroxidized lipids reacted with TBA to form a pink compound, which was measured using a spectrophotometer at 532 nm. A calibration curve was generated using malondialdehyde as the standard to calculate TBARS concentration. The results were expressed in µmol/mL of plasma or µmol/g of tissue.

### 2.4. Histopathology and Immunohistochemistry (IHC)

Sixty days after surgery, samples of BC, heart, liver, and kidneys were evaluated using histopathological techniques. The tissue slices were stained with hematoxylin and eosin staining (HE) and examined under light microscopy.

The samples were evaluated in normal or altered tissue and classified as degenerative, necrotic, or inflammatory in four categories: absent (normal tissue), mild (until 15% of the tissue was compromised), moderate (until 50% was compromised tissue), or severe/intense (up to 75% of the sample compromised). In the inflammatory case, foreign body giant cells, fibrosis, and vascularization were compiled as explained above.

The inflammatory process was classified as: neutrophilic, with a predominance of neutrophils without signs of infection; mononuclear, with a predominance of lymphocytes, plasmocytes, monocytes, and macrophages; granulomatous, with the presence of foreign body giant cells granuloma; pyogranulomatous, with the presence of neutrophils, macrophages, and giant cells in the same sample; and eosinophilic, with a predominance of eosinophils at the inflammatory site.

The antigen retrieval for CD3, CD20, and F4/80 antibodies was made with TRIS EDTA pH 9.0 in Pascal (Dako Denmark A/S), and collagen I (CI), collagen III (CIII), collagen IV (CIV), alpha smooth muscle actin (α-SMA) with citrate pH 6.0 in Pascal (Dako Denmark A/S). The next reactions used Envision Flex High pH Link K 800021-2 (Dako), following the data sheet instructions. The slices were counterstained with Harris hematoxylin and evaluated under light microscopy.

The negative control was performed by replacing the primary antibody with the antibody diluent, while the positive control used 4 µm sections of rat lymph node subjected to the same procedures.

Lymphocytes CD3 type were detected with Dako polyclonal antibody (A0452), 1:500 dilution; CD20 lymphocytes were marked with SpringBioscience polyclonal antibody, 1:200 dilution; and for F4/80 antibodies, the Abcam monoclonal antibody (ab300421), 1:5000 dilution, was used.

Collagens I, III, and IV were marked with Abcam polyclonal antibody (ab254113), 1:200 dilution; Abcam polyclonal antibody (ab7778), 1:300 dilution; and Abcam polyclonal antibody (ab6586), 1:300 dilution, respectively. Finally, alpha smooth muscle actin (α-SMA) was detected with Dako monoclonal antibody (M0851), 1:1000 dilution.

The reactions were evaluated as positive or negative accordingly the specifications of the manufacturers.

For lymphocytes CD3, CD20, and macrophages F4/80, all the cells with positive reactions were counted for each kind of cell. For the analysis of collagens, I, III, IV, and α-SMA, the staining reactions around the BC tissue were evaluated. The data were recorded in a Microsoft Excel spreadsheet.

### 2.5. Statistical Analysis

For the biochemical and oxidative stress markers, student’s *t*-test was applied to verify differences between the groups. Values of *p* < 0.05 were considered significant. The results were analysed using Statistica^®^ 8.0 and GraphPad Prism^®^ 5 software.

Histopathological evaluations used descriptive analysis for all assessed parameters. Results were expressed as percentages, frequencies, and means for each variable, and presented in tables and/or graphs.

For immunohistochemistry of antibodies CD3, CD20, and F4/80, due to the variation in the number of histological field readings per animal, the data were normalized and compared considering the total number of cells in each comparison. The following equation was used to standardize the data:

$$relative\;\%\;of\;cells\;per\;animal=\;\frac{\;(\;cell\;n{}^{\circ}\;per\;animal\times 100)}{total\;n{}^{\circ}\;of\;cells}$$

Data underwent logarithmic transformation and were assessed for normal distribution using the Shapiro–Wilk test and quantile-quantile (Q-Q) plots (of the residuals) [33].

Comparisons between the means of the implant (IG) and sham (SG) groups for CD3 and F4/80 cell types were conducted using an unpaired *t*-test. For CD20 lymphocytes, the Mann–Whitney test was used [33].

Comparisons of means between IG and SG for all three cell types (CD3, CD20, and F4/80) were performed using conventional ANOVA with Tukey’s adjustment and Welch’s ANOVA with Games–Howell adjustment, respectively. Statistical significance was set at *p* < 0.05. All analyses were conducted using GraphPad Prism (version 8.0.1).

## 3. Results and Discussion

**Figure 1:**
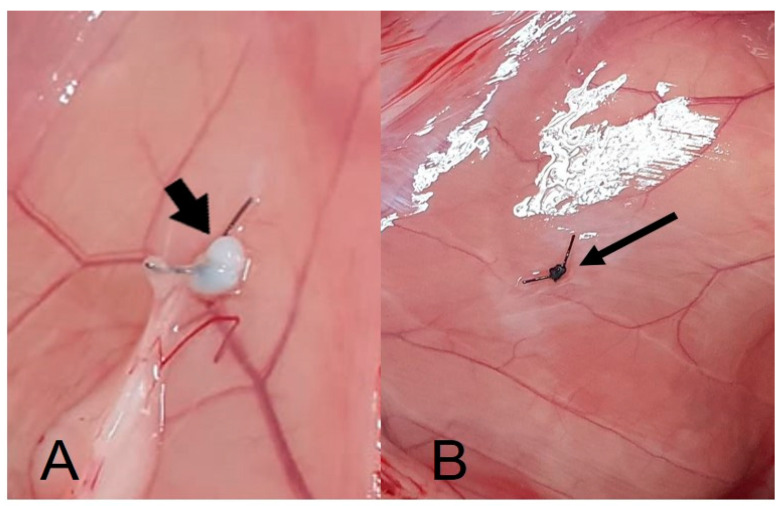
(**A**). Bacterial membrane implantation. The arrow indicates the membrane sutured to the animal’s peritoneum, 60 days after surgery. (**B**). The arrow indicates the nylon thread sutured in the peritoneum (sham), 60 days after surgery.

The animals tolerated the surgical procedures well, and the analgesic protocol was effective. Over the 60-day postoperative observation period, no complications were noted. Macroscopically, there were no visible changes indicating any adverse reaction to the BC and/or the nylon thread (Figure 1).

### 3.1. Oxidative Stress

**Figure 2:**
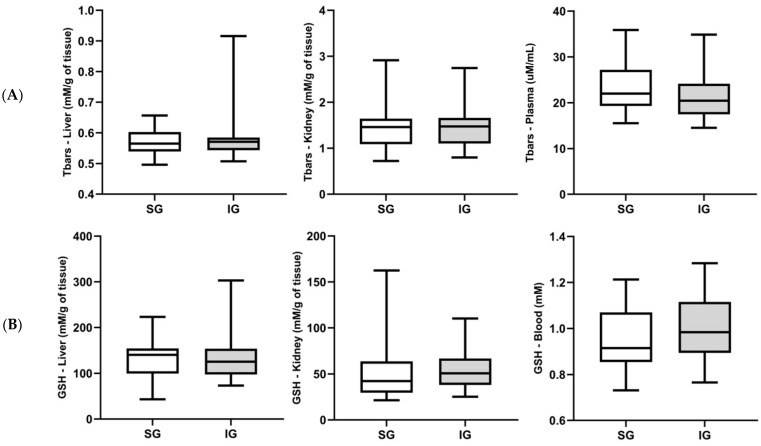
Concentration of (**A**) thiobarbituric acid reactive substances (TBARS) and (**B**) reduced glutathione (GSH) in liver, kidney, and plasma or blood, considering the sham group and the implanted group with the bacterial cellulose membrane. Data are expressed as mean ± standard deviation.

The results of TBARS and GSH are reported in Figure 2A,B, respectively. No statistically significant differences were observed between the implanted and sham groups in either blood or tissue samples (liver and kidney). This finding suggests that the use of the BC membrane did not induce lipid peroxidation, as indicated by TBARS, or a decrease in the antioxidant GSH.

**Figure 3:**
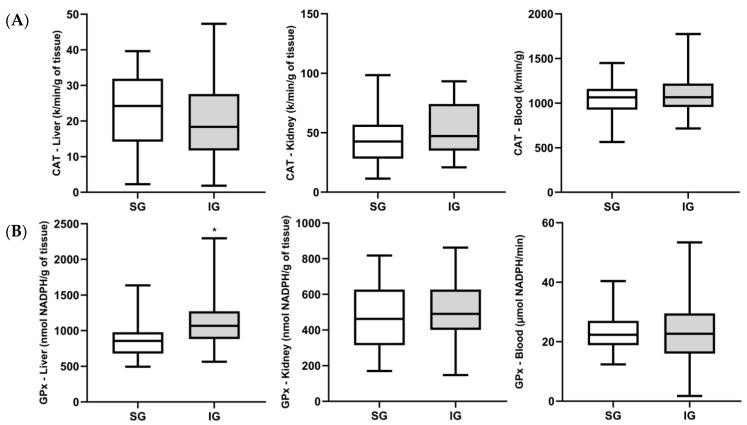
Activity of (**A**) catalase (CAT) and (**B**) glutathione peroxidase (GPx) in liver, kidney, and blood, considering the sham group and the implanted group with BC. The data are expressed as mean ± standard deviation. Note: * *p* < 0.05 compared to the sham group.

Regarding the antioxidant enzymes, the activities of catalase (CAT) and glutathione peroxidase (GPx) are reported in Figure 3A,B, respectively. An increase in GPx activity was observed in the liver of rats implanted with the BC membrane compared to the sham group (*p* < 0.05). On the other hand, GPx activity in the implanted group remained similar to the sham group in the kidney and blood, as did CAT activity in both tissues and in the blood.

The excessive production of free radicals and reactive oxygen and nitrogen species (ROS and RNS) results in a condition known as oxidative stress, which can cause damage to lipids, proteins, and DNA [34]. In this study, based on the evaluated markers, no lipid damage was observed, as TBARS concentrations remained similar in both groups. However, there was a change in the activity of one of the evaluated enzymes. Given that only one parameter was altered in a specific organ, we suggest the possibility of hepatic toxicity via an oxidative stress mechanism, but at a molecular level. The bacterial cellulose membrane, being a foreign material to the organism, may have induced an increase in the production of ROS and RNS. Another possibility is that these reactive species may have been produced in response to chronic inflammation [35].

Chronic oxidative stress can result from long-term exposure to factors such as radiation, pollution, inappropriate diet, or exposure to xenobiotics, which can damage and alter hepatic cells. The observed increase in GPx activity may represent an adaptive cellular response to continuous oxidative stress. Another plausible explanation for the elevated GPx activity in the implanted group could be the extended duration of anesthesia required for the surgery, given that the drugs used are metabolized hepatically.

### 3.2. Biochemical Tests

**Figure 4:**
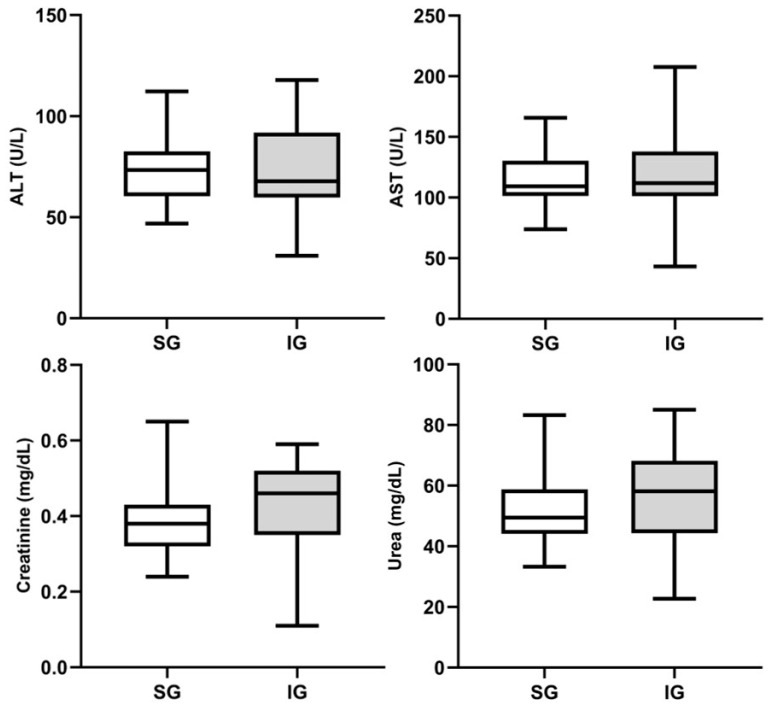
Biochemical parameters between implanted and sham groups, considering liver markers ALT (alanine aminotransferase) and AST (aspartate aminotransferase) and kidney markers creatinine and urea. Data are expressed as mean ± standard deviation.

The results of liver and kidney functions are presented at Figure 4. The results demonstrate the proximity between the absolute mean values of the implanted and sham groups in the measured parameters (ALT, AST, creatinine, and urea). Similarly, the statistical test (Student’s *t*-test) showed *p*-values > 0.05 in all analyses between the groups, indicating no statistical differences, with similarity in means, underscoring the safety of using BC as a biomaterial. Comparatively, Jeong et al. (2010) evaluated male mice that received an intraperitoneal injection of BC nanofibers, with subsequent biochemical analyses of ALT, AST, and creatinine showing no significant alterations between the animal groups, corroborating our findings. These facts highlight the non-toxicity of BC [36].

**Figure 5:**
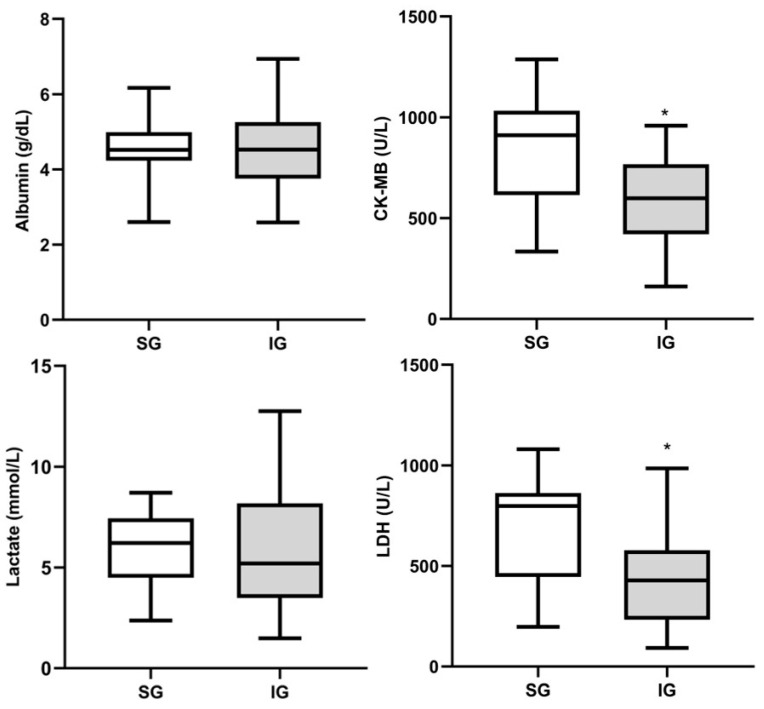
Box-plot of biochemical parameters between sham and implanted groups, considering albumin, creatine kinase MB (CK-MB), lactate, and lactate dehydrogenase (LDH). Data are expressed as mean ± standard deviation. Note: * *p* < 0.05 compared to the sham group.

Serum results for cardiac and inflammatory markers can be observed at Figure 5. The results of albumin and lactate denote similarity in the mean values between the implanted and sham groups. On the other hand, CK-MB and LDH show statistical significance, with IG exhibiting reduced serum mean compared to SG (*p* < 0.05). The sham group did not receive the peritoneal bioimplant but underwent the entire surgical procedure. This group exhibited higher serum mean compared to the implanted group, which received the BC membrane implant. The data support the safety of using BC as a biomaterial since the IG showed lower serum means in cardiac and inflammatory assessment parameters, even though both groups were subjected to the same surgical procedure. The analytes CK-MB and LDH revealed elevated serum means in both groups; however, SG exhibited an even higher serum mean than IG.

Expanding on the application of BC in veterinary medicine, Zharikov et al. (2018) conducted tests by implanting a bacterial nanocellulose membrane in five dogs, aiming to repair inguinal hernias. The study was divided into two phases. The first phase involved analysing results at 14 days after the anterior abdominal wall received the fixed bacterial nanocellulose membrane. The second phase involved a comparative analysis at 60 days following the simulation of the hernia defect, where the midline of the abdomen was incised, and grafts of bacterial nanocellulose and polypropylene mesh were applied. Subsequent visual and histological analyses showed that the grafts demonstrated biointegration with the tissue, and no infection related to the use of bacterial nanocellulose was found. These findings, combined with the results of this research, support the use of BC as a new biomaterial to be used within the peritoneal cavity [9].

### 3.3. Histopathology

Heart, liver, and kidneys did not present significant anatomopathological changes, corroborating the results found in biochemical and oxidative stress tests.

**Figure 6:**
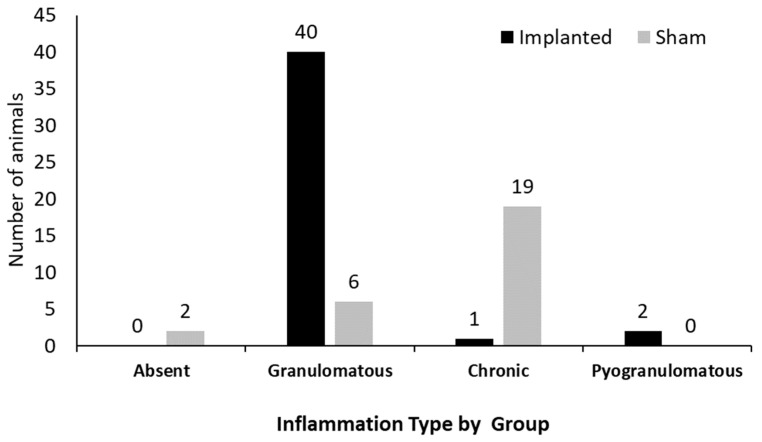
Type of inflammatory infiltrate observed in the implanted and sham groups, respectively.

**Figure 7:**
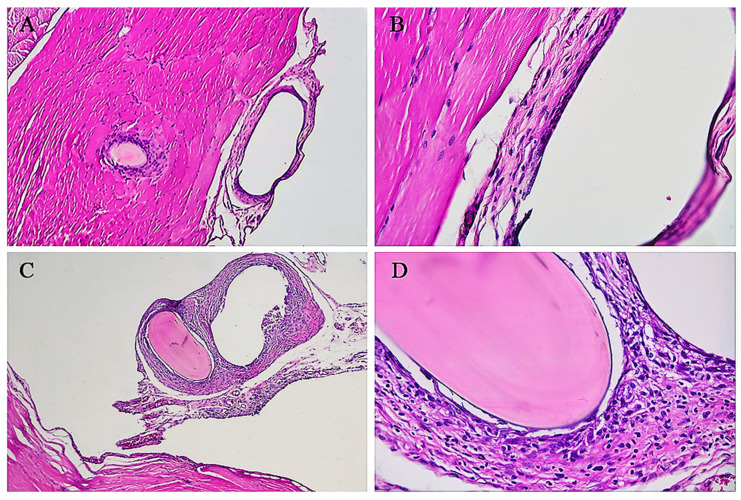
Histopathological grades of inflammation observed in animals in the sham group. (**A**). Grade 1 (mild) inflammatory process at 40× magnification. HE, 4× objective. (**B**). Detail of the inflamed region at 400× magnification. HE, 40× objective. (**C**). Grade 2 (moderate) inflammation at 40× magnification. HE, 4× objective. (**D**). Detail of the inflamed region at 400× magnification. HE, 40× objective.

The predominant type of inflammation in the implanted group was a granulomatous, foreign body type, occurring in 40 (93%) animals, followed by pyogranulomatous in two (5%) animals and chronic mononuclear inflammation in one (2%) of the rats. In the sham group, 19 (70.5%) individuals presented chronic inflammation of the mononuclear type, and six (22%) of the granulomatous type, whereas two (7.5%) animals did not present signs of an inflammatory process (Figure 6 and Figure 7) (Supplementary Material).

The inflammatory cells observed in both groups were lymphocytes, macrophages, and foreign body giant cells. Two pyogranulomatous processes were observed in the IG, probably due to some individual predisposition or some intra-operative factor.

Figure 7 illustrates the main inflammatory alterations observed in the sham group.

**Figure 8:**
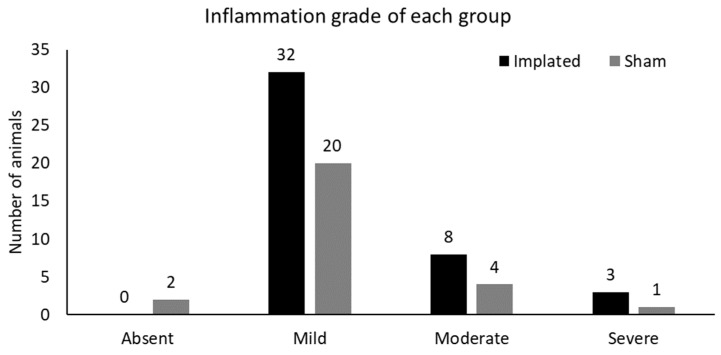
Degree of inflammatory infiltration observed in the implanted groups and sham groups.

Regarding the intensity (degree) of inflammation, in the implanted group, 32 (74%), 8 (19%), and 3 (7%) were classified as mild, moderate, and intense, respectively. In the sham group, 2 (7.4%) animals were classified as having zero (absence) inflammation, 20 (74%) as having a slight degree, 4 (15%) as having a moderate degree, and 1 (3.6%) as having an intense degree of inflammation (Figure 7 and Figure 8).

In the implanted group, 75% and 19% of the animals had discrete (degree I) and moderate (degree II) inflammatory reactions, respectively. This differed from a previous study in which even at 90 days post-operation, there was a predominance of moderate inflammatory reactions. One hypothesis for this difference may be due to location of implantation and the animal species, since BCs were implanted in the subcutaneous tissue of mice in the previous study, instead of in the peritoneum of rats, as in this case [37].

Our findings are consistent with the results of Zharikov et al. (2018), who also evaluated inflammatory responses in a similar context. In their study, conducted with dogs, they reported the presence of mononuclear cells, including lymphocytes, macrophages, and giant cells, 60 days post operatively [9]. This observation aligns with our study’s results where similar inflammatory cell types were detected in response to BC membrane implantation. These similarities suggest that the inflammatory response to BC membranes may be consistent across different animal models

Regarding the presence of foreign body giant cells, in the sham group, giant cells were observed in six (22%) cases, since granulomatous inflammations were present in this group. In the implanted group, 42 (98%) had giant cells. It is worth mentioning that foreign body giant cells are part of foreign body granulomatous inflammation, and this result is expected for this pattern of inflammation (Figure 7).

This same cell type has also been found in other studies, such as in Maia et al., (2018) and Zharikov et al. (2018), even though these studies were conducted with different animal species and tissues. In this sense, these findings reinforce our results from the point of view of inflammatory reaction and the biocompatibility of the BC [9,38].

The vascularization in the individuals of the sham group was as follows: 2 (7,0%) animals of grade zero (absent), 17 (63%) mild, 4 (15%) moderate, and 4 (15%) intense. Regarding the IG, the numbers were: zero (0%), 23 (53.5%), 11 (25.5%), and 9 (21%), for grades zero to three, respectively (Figure 7).

**Figure 9:**
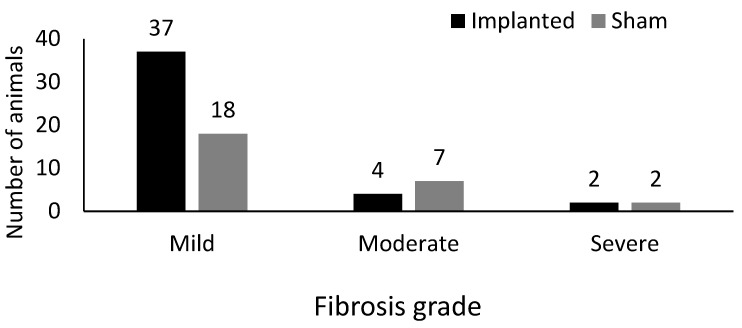
Degree of fibrosis observed in implanted groups and sham groups.

The degrees of fibrosis for the animals of the sham group were: zero (0%), 18 (66.5%), 7 (26%), and 2 (7.5%), of degrees zero to three, respectively. In comparison, in the IG group, it was observed: zero (0%), 37 (86%), 4 (9%), and 2 (5%) animals for grades zero to three, respectively (Figure 7 and Figure 9).

**Figure 10:**
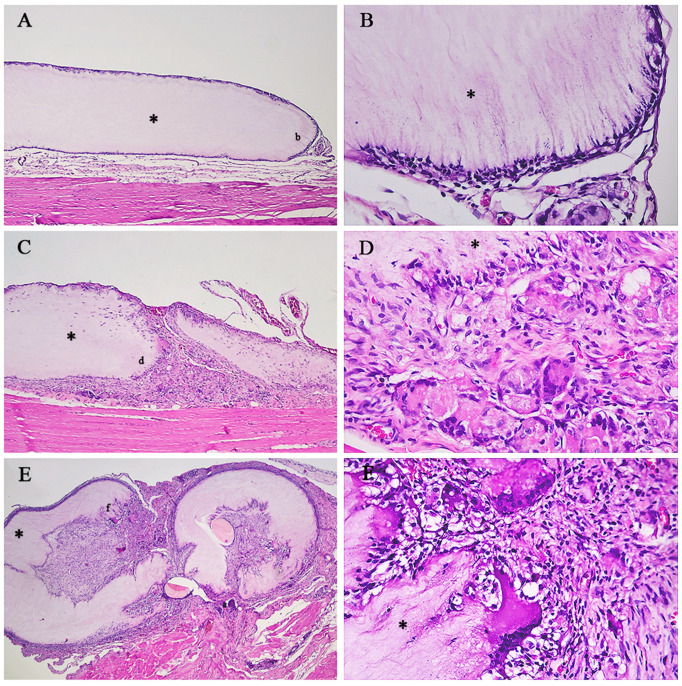
Histopathological grades of the inflammations observed in animals in the group implanted with the bacterial cellulose membrane (*). (**A**). Grade 1 inflammatory process at 40× magnification. HE, 4× objective. (**B**). Detail of the inflamed region at 400× magnification (b). HE, 40× objective. (**C**). Grade 2 inflammation, that should be observed in comparison to A. HE, 4× objective. (**D**). Higher magnification of the same region (d). HE, 40× objective. (**E**). Membrane implantation site with grade 3 inflammation. HE, 4× objective. (**F**). 400× magnification of grade 3 inflammation (f). HE, 40× objective.

The degrees of inflammation observed in the histopathological analyses of the animals in the group experimentally implanted with bacterial membranes are detailed in Figure 10.

Although it was not one of the aims of this study, the presence of adhesions with the abdominal viscera was minimal, which is desirable for implants of this nature.

About biocompatibility, it was observed that the BC causes a granulomatous reaction of the foreign body type, with a predominance of discrete inflammatory reactions (32/43—74%—grade I). Despite not being entirely desirable, this fact does not exclude the possibility of the use of BCs as a bioimplants, as the inflammatory process tends to decrease with time and is influenced by the tissue that receives the BC [9,23,38]. In addition, it encourages future studies aimed at producing BC-based medical biodevices.

BC can also accelerate the epithelialization process and prevent infections. Furthermore, biocomposites of these membranes have the potential to regulate cell adhesion, which is an important feature for scaffolds and grafts [22,39,40,41,42].

After 12 weeks of bacterial cellulose implantation in Wistar rats, it was observed that no fibrotic capsules or giant cells were detectable by microscopy, indicating that no foreign body reaction occurred. Additionally, macroscopically, no redness, swelling or exudate developed around the implantation sites [8].

Lee et al. (2015) compared collagen and bacterial cellulose membrane grafts in rats. The response of BC membrane showed significantly higher mechanical properties than collagen membrane, such as wet tensile strength. Bacterial cellulose (BC) membrane also represented a three-dimensional multilayer structure, cross-linked by nanofibers with 60% porosity. In the in vivo study, the grafted BC membrane did not induce an inflammatory response and maintained an appropriate space for bone regeneration [43].

Nimeskern (2013) and Martínez (2014) investigated the use of bacterial cellulose in ear cartilage tissue engineering in vitro and in vivo in rabbits, respectively. These authors concluded that BC has the ability to achieve mechanical properties of relevance for auricular cartilage replacement, due to its similarity to the auricular cartilage in terms of mechanical strength and host tissue response [20,44].

Pértile et al. (2012) implanted BC in the subcutaneous tissue of mice and stated that it is biocompatible [31]. Zharikov et al. (2018) studied five dogs to investigate whether an anterior abdominal wall defect could be repaired using compact (99.9%) moist BC membranes, produced by the symbiotic culture of *Medusomyces gisevii*. The abdominal wall defect was simulated by midline laparotomy and the membrane was then fixed. The materials were collected at 14 and 60 days postoperatively. Bacterial cellulose (BC) exhibited good fixation to the anterior abdominal wall to form, on the 14th day, a loose fibrin capsule around the membrane. Repair processes were observed at the BC site 60 days after surgery, generating new and stable conjunctive tissue elements (macrophages, giant cells, fibroblasts, fibrin), neo capillaries, and collagen elements. This generated a dense postoperative scar whose intraperitoneal adhesions to the bowel loops were insignificant [9].

The presence and intensity of fibrosis can lead to regeneration or rejection of the biomaterial. In addition, the microenvironment of the region in which the membrane will be placed also plays an important role in the acceptance or rejection of the BC [45,46].

### 3.4. Immunohistochemistry (IHC) CD20, CD3 e F4/80

This study seems to be the first one regarding typing and quantifying the lymphocytes and macrophages that participate in the inflammatory process related to BC.

**Figure 11:**
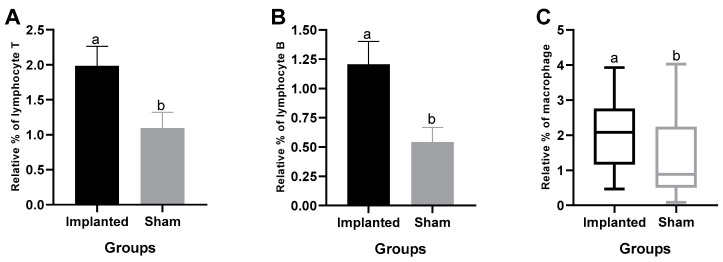
(**A**,**B**) Mean (+/− SEM) percentage of CD3 and CD20 lymphocytes, respectively, between the implanted and sham groups. (**C**) Median (Max—Min) percentage of macrophages between the implanted and sham groups. Statistical differences (*p* < 0.05) are indicated within each group by lowercase letters.

Cell counts for positive CD3 (T lymphocytes), CD20 (B lymphocytes), and F4/80 (macrophages) were performed in all animals, both in the implanted and the sham group. For the CD3 antibody, the mean +/− standard deviation was 1.987 (+/− 1.663) in the implanted group and 1.095 (+/− 1.153) in the sham group (Figure 11A).

For B lymphocytes (CD20), the mean +/− standard deviation was 1.207 (+/− 0.9788) in the implanted group and 0.5422 (+/− 0.6211) in the sham group. Since the data did not meet the normal frequency distribution, the medians (Q1-Q3) were used, with the median for IG being 0.9466 and for SG being 0.2151 (Figure 11B) (Supplementary Material).

The statistical analysis of the macrophage count (F4/80) revealed that the mean +/− standard deviation was 2.047 (+/− 0.9449) in the implanted group and 1.406 (+/− 1.117) in the sham group. Therefore, there was a significant difference in the number of macrophages between the IG and SG groups (*p* < 0.05) (Figure 11C).

**Figure 12:**
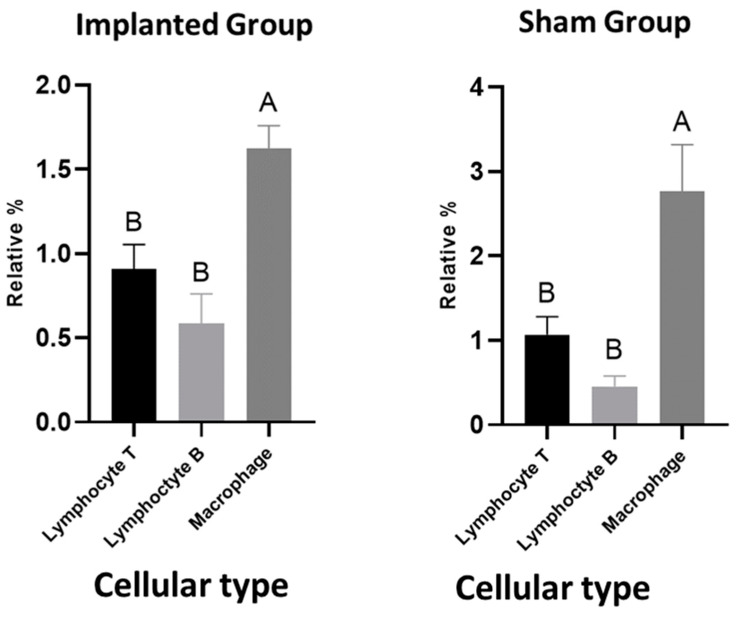
Mean (+/− SEM) percentage of T and B lymphocytes, as well as macrophages, in the implanted group and sham group. Statistical differences are indicated by different uppercase letters (*p* < 0.05).

When comparing the results obtained intragroup for the three antibodies (CD3, CD20 and F4/80), the following averages were obtained in IG: CD3 0.9092 (+/− 0.8741), CD20 0.5834 (+/− 0.9511) and F4/80 1.624 (+/− 0.7498). This shows a significant difference in the number of macrophages versus T and B lymphocytes; however, there was no difference between the types of lymphocytes (*p* < 0.05) (Figure 12).

In the sham group, the averages were: CD3 1.068 (+/− 1.098), CD20 0.4549 (+/− 0.6545) and F4/80 2.760 (+/− 2.847), with a significant difference between the number of macrophages and both types of lymphocytes. There was no difference between the number of types of lymphocytes (*p* < 0.05) (Figure 12).

**Figure 13:**
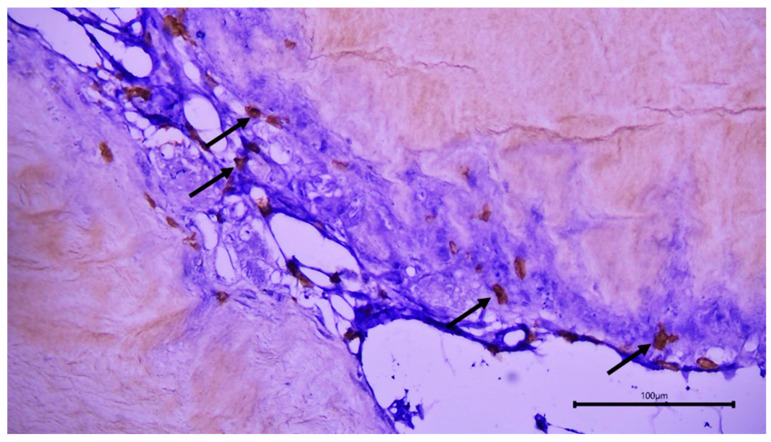
BC implantation site. Immunohistochemical staining for CD20. Positive staining of B lymphocytes around the membrane (arrows).

**Figure 14:**
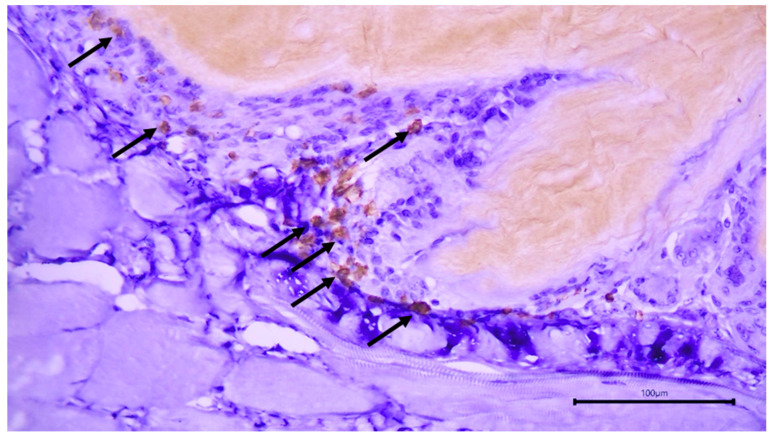
BC implantation site. Immunohistochemical staining for CD3. Positive staining of T lymphocytes around the membrane (arrows).

The immunohistochemical staining pattern of the two antibodies can be seen in Figure 13 and Figure 14.

### 3.5. Immunohistochemistry (IHC) Type I; III and IV Collagens and α-SMA

Similarly to the immunohistochemistry of inflammatory cells, this seems to be the first study to identify the types of collagens and vascularization in the reaction process generated by BC.

There was no positive staining for collagen IV in all samples tested, as required by the immunohistochemistry protocol. Positive and negative controls were included in the ztest batteries and were duly verified, confirming the reactions described above.

The analysis of reactivity for collagen I (CI) revealed positive staining in all samples when the internal control was verified.

**Figure 15:**
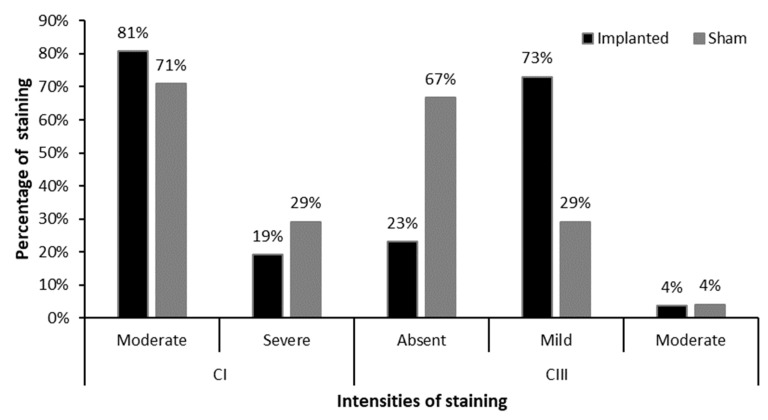
Percentage of staining and its respective intensities for collagens I and III in IG and SG, respectively.

**Figure 16:**
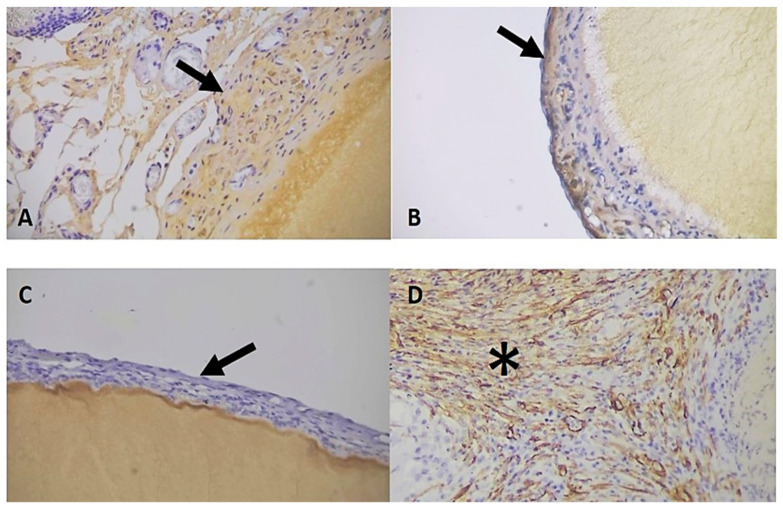
Degrees of staining for the collagen types and α-SMA in the animals of the implanted group. (**A**). Observe moderate staining for CI around the membrane (arrow) (**B**). Observe discrete staining for CIII around the membrane (arrow) (**C**). Observe absence of staining for CIV around the membrane (arrow) (**D**). Observe intense staining of myofibroblasts and blood vessels around the membrane (*). IHC for CI, CIII, CIV, and α-SMA respectively at 400× magnification, 40× objective.

**Figure 17:**
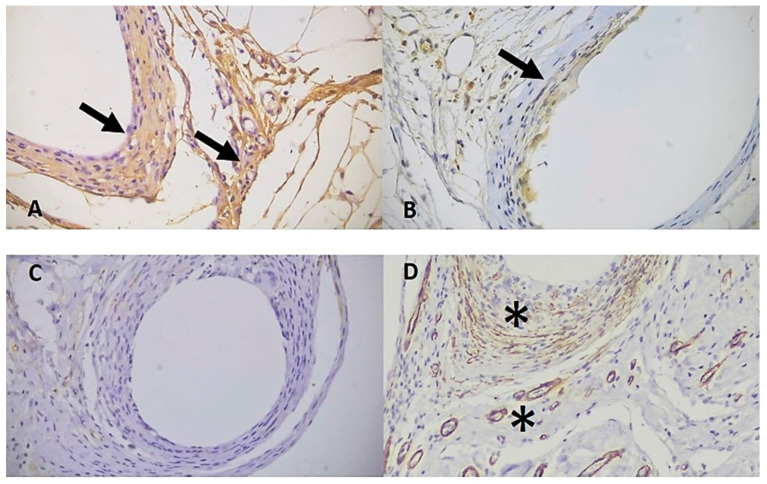
Degrees of staining for collagens types and α-SMA in animals in the sham group. (**A**). Note intense staining for CI (arrows). (**B**). Note discrete staining for CIII (arrow). (**C**). Note absence of staining for CIV. (**D**). Note intense staining of myofibroblasts and blood vessels around the suture point (*). IHC for CI, CIII, CIV and α-SMA at 400× magnification, 40× objective.

In the implanted group (IG) the immunohistochemistry for CI exhibited a moderate staining in 21 out of 26 animals (81%), while 5 (19%) showed intense staining (Figure 15 and Figure 16). In the sham group, 17 out of 24 animals (71%) demonstrated moderate staining for CI, and 7 (29%) displayed intense staining (Figure 15 and Figure 17).

For collagen III (CIII) analysis, out of 26 animals of the IG, 6 (23%) did not present staining for the antibody, while 19 (73%) presented mild staining and 1 (4%) moderate staining (Figure 15 and Figure 16). In 16 (67%) animals in the sham group, there was no staining in 16 samples (67%), while 7 (29%) presented mild staining and 1 (4%) moderate staining, totalling 24 samples (Figure 15 and Figure 17). This result supports that CI and CIII are respectively at 60 days after the implantation the major type of collagen around BC.

**Figure 18:**
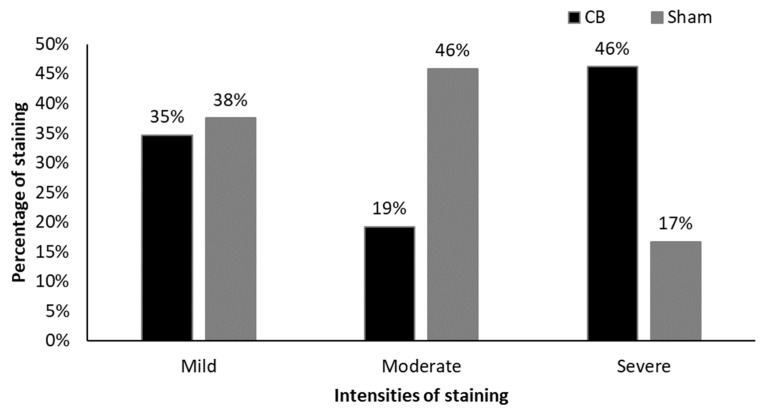
Percentage of staining and its respective intensities for α-SMA in the IG and SG, respectively.

In the alpha smooth muscle actin (α-SMA) analysis, for the evaluation of late vascularization around the membrane, the intense degree was more frequent in the IG, corresponding to 12 (46%) animals, followed by 9 (35%) mild and 5 (19%) moderate staining (Figure 16 and Figure 18).

In the sham group the alpha smooth muscle actin (α-SMA) analysis showed moderate degree as the most frequent, present in 11 (46%) animals, followed by 9 (38%) mild and 4 (17%) intense (Figure 17 and Figure 18).

It can be noted that even after 60 days of the surgical procedure, vascularization in both groups remained present in a moderate to intense form in most of the samples analysed.

## 4. Conclusions

Overall, both the biochemical and oxidative stress markers at the systemic level suggest the safety of bacterial cellulose membrane for use as an intraperitoneal bioimplant. Bacterial cellulose membrane induces a local granulomatous inflammatory response, typical of a foreign body reaction, but predominantly of mild intensity, which further supports its use as a biomaterial for intraperitoneal implantation. The quantity of macrophages (F4/80) was significantly higher than that of T lymphocytes (CD3) and B lymphocytes (CD20). Regarding collagen fibres, type IV collagen was absent, while type I and III were present. At 60 days post-operation, the vascularization around the membrane varied from moderate to intense when assessed by smooth muscle alpha-actin staining. Based on these findings, BC membrane is safe for use as a peritoneal implant. More studies are necessary in bioengineering field to surface modifications of the BC to improve the characteristics of biocompatibility.

## Data Availability

Research data may be shared under solicitation to the authors.

## Supplementary Materials

The following supporting information can be downloaded at: https://www.mdpi.com/article/10.3390/cimb46110697/s1. Table S1. Histopathological classification of the implanted animals and sham. Figure S1. (A) and (B) Mean (+/− SD) percentage of CD3 and CD20 lymphocytes, respectively, between the Implanted and sham groups. Statistical differences (*p* < 0.05) are indicated within each graph, by lowercase letters.
