# Supplementary material for: Bacterial Cellulose Membrane Experimentally Implanted in the Peritoneum of Wistar Rats—Inflammatory Immunoreactivity and Oxidative Stress
Source: Curr Issues Mol Biol. 2024 Oct 22;46(11):11729–48. doi: 10.3390/cimb46110697 (PMC11592940; doi:10.3390/cimb46110697)
Supplement: Supplementary file 1 [file cimb-46-00697-s001.zip › cimb-3220395-supplementary.pdf]

Supplementary Materials:

Table S1. Histopathological classification of the implanted animals and sham.

N	ID	Sex	Inflammation type	Degree	GC	Fibrosis	V.
1	IG01	M	Granulomatous	1	1	1	3
2	IG02	M	Granulomatous	1	1	1	3
3	IG03	M	Granulomatous	1	1	1	3
4	IG04	M	Granulomatous	1	1	1	3
5	IG05	F	Granulomatous	1	1	1	2
6	IG06	M	Granulomatous	2	1	2	3
7	IG07	M	Granulomatous	1	1	1	1
8	IG08	M	Granulomatous	3	1	2	3
9	IG09	M	Pyogranulomatous	3	1	3	3
10	IG10	M	Granulomatous	1	1	1	2
11	IG11	M	Granulomatous	1	1	1	2
12	IG12	M	Granulomatous	2	1	1	3
13	IG13	M	Granulomatous	2	1	1	3
14	IG14	M	Granulomatous	1	1	1	2
15	IG15	M	Granulomatous	1	1	1	2
16	IG16	M	Granulomatous	1	1	1	2
17	IG17	M	Granulomatous	1	1	1	2
18	IG18	M	Granulomatous	1	1	1	1
19	IG19	M	Granulomatous	2	1	1	1
20	IG20	M	Granulomatous	1	1	2	2
21	IG21	M	Granulomatous	1	1	1	2
22	IG22	M	Granulomatous	2	1	1	1
23	IG29	F	Granulomatous	1	1	1	1
24	IG30	F	Granulomatous	1	1	1	1
25	IG31	F	Granulomatous	1	1	1	1
26	IG34	F	Granulomatous	1	1	1	1
27	IG38	F	Granulomatous	1	1	1	2
28	IG39	F	Granulomatous	1	1	1	1
29	IG41	F	Pyogranulomatous	3	1	3	2
30	IG42	F	Granulomatous	1	1	1	1
31	IG43	F	Granulomatous	1	1	1	1
32	IG44	F	Granulomatous	1	1	1	1
33	IG45	F	Granulomatous	2	1	1	1
34	IG46	F	Granulomatous	1	1	1	1
35	IG48	M	Granulomatous	1	1	1	1
36	IG49	M	Granulomatous	2	1	2	1
37	IG50	M	Chronic	1	0	1	1
38	IG54	F	Granulomatous	1	1	1	1

39	IG55	F	Granulomatous	1	1	1	1
40	IG56	F	Granulomatous	2	1	1	1
41	IG59	F	Granulomatous	1	1	1	1
42	IG61	M	Granulomatous	1	1	1	1
43	IG62	M	Granulomatous	1	1	1	1
44	SG23	F	Chronic	1	0	2	1
45	SG24	F	Absent	0	0	1	1
46	SG25	F	Chronic	1	0	2	2
47	SG26	F	Chronic	1	0	2	0
48	SG27	M	Chronic	1	0	1	1
49	SG28	M	Chronic	1	0	1	1
50	SG32	M	Granulomatous	1	1	1	1
51	SG33	M	Granulomatous	1	1	1	1
52	SG35	M	Chronic	1	0	3	3
53	SG36	M	Chronic	3	0	2	1
54	SG37	M	Chronic	2	0	1	1
55	SG40	M	Chronic	1	0	2	1
56	SG47	M	Chronic	1	0	1	2
57	SG51	M	Granulomatous	1	1	1	1
58	SG52	M	Chronic	1	0	1	3
59	SG53	M	Granulomatous	2	1	2	3
60	SG57	F	Granulomatous	1	1	1	1
61	SG58	F	Chronic	1	0	1	1
62	SG60	F	Chronic active	1	0	1	1
63	SG63	F	Chronic	1	0	1	1
64	SG64	F	Chronic	2	0	1	3
65	SG65	F	Absent	0	0	1	1
66	SG66	F	Chronic	1	1	3	1
67	SG67	F	Chronic	1	0	1	2
68	SG68	F	Chronic	1	0	1	0
69	SG69	F	Chronic	2	0	2	2
70	SG70	F	Granulomatous	1	1	1	1

* N = animal number. ID = animal identification according to the Group (G), where I is the implanted and S is sham. GC = Giant Cell. V = Vascularization.

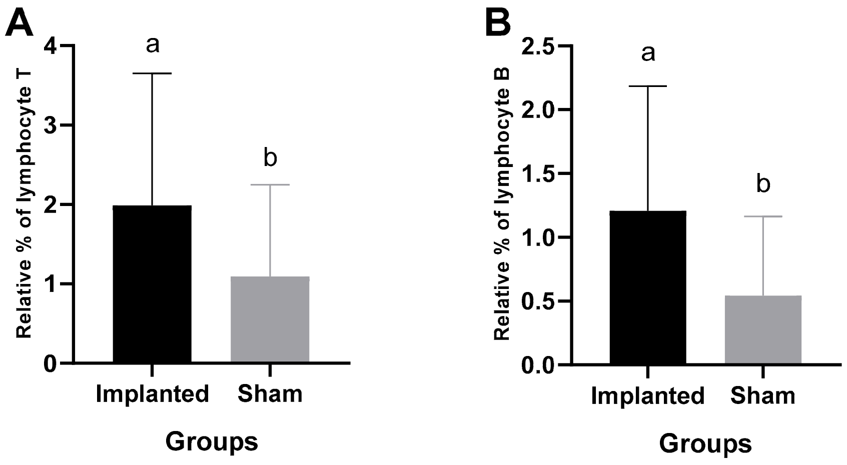

Figure S1. (A) and (B) Mean (+/− SD) percentage of CD3 and CD20 lymphocytes, respectively, between the Implanted and sham groups. Statistical differences (p < 0.05) are indicated within each graph, by lowercase letters.
